# Features and quality metrics datasets for video coding in DASH

**DOI:** 10.1038/s41597-024-03507-6

**Published:** 2024-06-26

**Authors:** Francisco Micó-Enguídanos, Andoni Salcedo-Navarro, Miguel Garcia-Pineda, Juan Gutiérrez-Aguado

**Affiliations:** Universitat de València, Departament d’Informàtica, Av. de la Universitat s/n, Burjassot, 46100 València Spain

**Keywords:** Technology, Engineering

## Abstract

Nowadays, the majority of media Internet traffic consists of H.264-encoded streaming videos due to its high compatibility. One of the most popular streaming technology used to deliver videos over Internet is Dynamic Adaptive Streaming over HTTP (DASH). It transmit the video as a sequence of independent short video segments tailored to the receiver’s limitations (related to several factors such as available bandwidth or resolution on reception), aiming to enhance the Quality of Experience. In this paper, we present two types of datasets created from 4065 video segments of 2 seconds. The first type consists of extracting features related to color, space, and time from the segments across different vertical resolutions (240, 360, 480, 720, 1080, 1440 and 4K). The second one includes several quality metrics obtained from the same segments when they are encoded with different compression levels in the different resolutions.

## Background & Summary

Sandvine’s 2023 Global Internet Phenomena Report (https://www.sandvine.com/global-internet-phenomena-report-2023) shows that the global Internet traffic volume increased by 23%, due in large part to streaming video usage and continued growth in traffic across app categories. According to this report, video usage grew by 24% in 2022, now representing the 65% of all internet traffic. For the first time, Netflix replaced YouTube as the individual app generating the most traffic, with TikTok, Disney+, and Hulu among the top-10. The first streaming systems emerged in the 1990s, and since then, technologies supporting these systems have evolved significantly with advancements in technology and network connections. Today’s streaming platforms have benefited from these advancements and have themselves further contributed to the state of the art in streaming technology.

H.264/MPEG-4 AVC (https://www.itu.int/rec/T-REC-H.264) is the most widely used video codec in several types of transmissions. According to the 6th Annual Bitmovin Video Developer Report (https://bitmovin.com/resources/), surveyed industry professionals use it in production of Live and Video On Demand streaming at 78% and 85% respectively. H.264 is currently used in Blue-Ray movies, streaming services, satellite broadcasting, and even digital terrestrial television. It is known for its excellent compatibility at all levels and strong support for real-time streaming.

To deliver the videos to the users with a good level of Quality of Experience (QoE), the predominant video technology used by most streaming platforms is HTTP Adaptive Streaming (HAS)^1^, being DASH the international standard. In HAS, video content is divided into small fragments, known as segments, which typically range from one to ten seconds in duration. To provide viewers with different quality options, several representations of the video are created at various quality levels. Each segment is encoded or transcoded using different resolutions, bit rates, codecs, and other parameters. The information about the generated content, such as video profiles, metadata, codecs, bandwidth, server IP addresses, and download URLs, is described in a Media Presentation Description (MPD) manifest file. Both the MPD and the segments are stored on standard HTTP servers. This allows each client to dynamically select the segments based on the available network bandwidth and other factors, resulting in a smoother streaming experience for the end user.

During the encoding process, as the required bitrate decreases (consequently generating small final video size), artifacts, such as blocks and bluring, are more visible. In this context, reliable video quality assessment is essential to ensure the quality of the video service provided and to improve the quality of the end-user experience. The Video Quality Assessment (VQA) process can use a wide variety of metrics, with different levels of performance and complexity^2^. These metrics can be classified as subjective and objective. Subjective assessment is often regarded as the most reliable and accurate method to assess video quality. However, these tests are costly, time-consuming, and cannot be directly integrated into automated decision-making systems. As a result, objective assessment methods have gained popularity due to their speed and scalability.

The main objective of this work is to present to the scientific community an extensive dataset of video characteristics and objective quality values for different resolutions with 45 Constant Rate Factors (CRF), ranging from 7 to 51. The CRF encoding mode dynamically adjusts the bit rate throughout the video to attain a quality level. Lower CRF values result in higher video quality, while higher CRF values may compromise quality to achieve smaller file sizes.

To date, there are several DASH-oriented datasets, but they differ in the nature of the data provided. The Multi-Codec DASH Dataset^3^ (MCDD) consider 10 videos (3 of them with 60 seconds duration and the rest with 20 seconds duration). The created videos possess 4K resolutions and lower for 4 codecs and two types of segment duration (2 and 4 seconds). The dataset offers the encoded videos and the MPD files. Another work of the same authors is Multi-Codec Ultra-High Definition 8K MPEG-DASH Dataset^4^ (MCDD8K). This work presents a dataset of 10 videos (8 with 4K resolutions and 2 with 8K resolution) with durations around 200 seconds. The videos were encoded with 4 codecs at various resolutions and with segment durations of 4 and 8 seconds. This dataset offers the encoded videos and the MPD files. Another recent dataset is LIVE-NFLX-II^5^. It analyzes several DASH videos of 1080p resolution and below, encoded with H.264/MPEG-4 AVC with 10 Quantization Parameter (QP) values. From these videos, Mean Opinion Score (MOS)^6^ values are obtained when there are changes in bandwidth, delays, jitter, losses, etc. In addition to these values, the Video Multi-Method Assessment Fusion (VMAF) (https://netflixtechblog.com/toward-a-practical-perceptual-video-quality-metric-653f208b9652) metric is extracted as an objective metric. The dataset offers the received videos under different network conditions and quality metrics. The main characteristics of these datasets and the one presented in this work are presented in Table 1.

**Table 1:** Comparison of datasets with our proposal.

Dataset name	Dataset content	Number of videos	Segment sizes (s)	Number of segments	Resolutions	Quality provided	Codecs	Encoding paremeter
MCDD	Encoded videos	10	2,4	240	11	No	4	19 Bitrates
MCDD8K	Encoded videos	10	4,8	890	12	No	4	15 Bitrates
LIVE-NFL-II	Received videos and QoE	15	25	420	6	Yes	1	10 QPs
This work	Features and quality	281	2	4065	7	Yes	1	45 CRFs

According to our study of the state of the art, there are no datasets that relate video features to objective quality metrics at different resolutions and quality levels. Besides, we think that this dataset could be very interesting for new researchers related to video encoding process for DASH environments as it can be used to train models with supervised learning techniques.

## Methods

This section outlines the entire process undertaken to generate the datasets containing the features and quality metrics derived from the multi-resolution videos encoded with several CRFs.

**Fig. 1:**
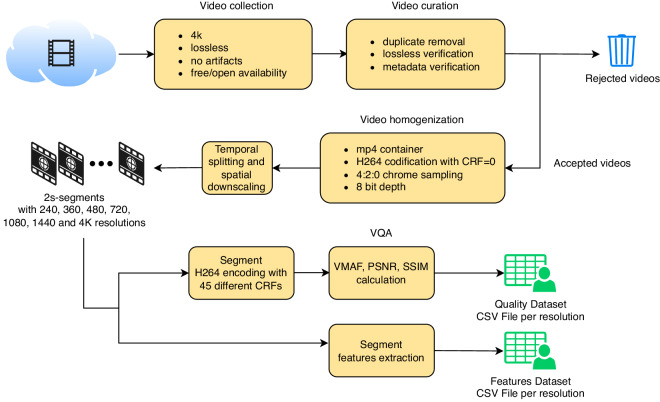
Main stages followed to build the dataset.

As depicted in Fig. 1, the initial step involved selecting 4K videos from publicly accessible databases on the Internet. Subsequently, we conducted a thorough screening of these videos to ensure they met our requirements: no duplicated videos, videos are in lossless format, and video and metadata concordance. Then, with the accepted videos we proceeded to homogenize the format, thereby facilitating the subsequent processing steps.

The subsequent stage involved splitting and downscaling the videos to create 2-second segments across various resolutions resulting in 4065 segments. Finally, we encoded all these segments using the H.264 encoder with 45 different values of CRF, thereby enabling us to calculate their respective quality metrics. Furthermore, we extracted the features from each segment and saved all the data in Comma Separated Values (CSV) files, categorized according to their respective resolutions.

Two platforms have been used for the creation of the datasets:

1. A virtual machine (VM) configured with 24 vCPUs and 48 GB RAM on a physical server with 128GB of RAM and 20 cores (40 threads). The VM is managed by the Kernel-based Virtual Machine (KVM) hypervisor (https://www.qemu.org).
2. A Kubernetes cluster that operates across three physical machines, each equipped with 128 GB of RAM and 36 cores (72 threads). The cluster consists of 9 VMs. The master node operates on a VM equipped with 16 GB RAM and 10 vCPUs, while the remaining VMs host the Kubernetes worker nodes. The virtual infrastructure is designed for high performance, utilizing SR-IOV NICs to provide direct network access to VMs, and is complemented by dedicated SSDs for each VM to ensure efficient and rapid data access. Docker (https://www.docker.com/) and Flannel (https://github.com/flannel-io/flannel) are utilized as the Container Runtime and Network Interfaces, respectively. Each encoding and quality computation is handled as an event and sent to RabbitMQ (https://www.rabbitmq.com). These events are then consumed asynchronously by event consumers, which are encapsulated in Kubernetes deployments.

The homogenization, splitting, downscaling, and feature extraction were performed for all videos or segments using the first platform. Encoding and VMAF calculation for segments with vertical resolutions less than or equal to 1080 were done using the first platform, while those for resolutions of 1440 and 4K were done using the second platform.

### Video collection

When gathering the 4K videos for constructing our datasets, we considered a set of constraints to prevent the introduction of errors or undesired effects during the encoding process and quality metrics calculations.

An essential constraint was that the videos needed to have a flawless 4K resolution, devoid of artifacts such as horizontal or vertical black bars. The following resolutions were considered valid 4K resolutions: 3840 × 2160 (4K UHD - Ultra High Definition, the prevailing 4K resolution in television and consumer media), 4096 × 2160 (DCI 4K - Digital Cinema Initiative, employed by the movie projection industry), and 4096 × 1714 or 4096 × 1744 (approximate CinemaScope resolution).

Another restriction we enforced was that the videos had to be lossless, as it was imperative for the quality metric calculations that such videos did not have undergone any kind of encoding that resulted in information loss.

Lastly, we exclusively selected video databases from which we could acquire videos free of charge. In certain instances, registration and the creation of a user account were necessary to access the videos, but no royalties needed to be paid.

The databases utilized for obtaining the videos to create the datasets are the following:

- The Consumer Digital Video Library^7^ (CDVL)
- The Audiovisual Technology (AVT) Group AVT-VQDB-UHD-1 video quality database^8^
- The Shanghai Jiao Tong University (SJTU) 4K video sequence dataset^9^
- The Xiph.org Test Media collections (XIPH, https://media.xiph.org)
- The Images and Video-Communications (IVC) UHD videos dataset^10^
- The Multimedia Computing and Machine Learning (MCML) 4K UHD video quality database^11^
- The YouTube User Generated Content (UGC) dataset^12^
- The open Ultra Video Group (UVG) dataset^13^
- The Tencent Video Dataset^14^ (TVD)
- The CableLabs 4K video dataset (CL, https://www.cablelabs.com/4k/)
- The Blender Foundation videos (BLEN, https://durian.blender.org/, https://mango.blender.org/)

**Fig. 2:**
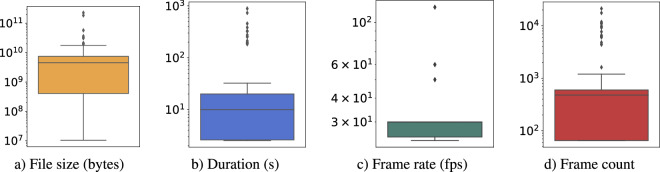
Statistics of the videos used to compute the datasets.

**Fig. 3:**
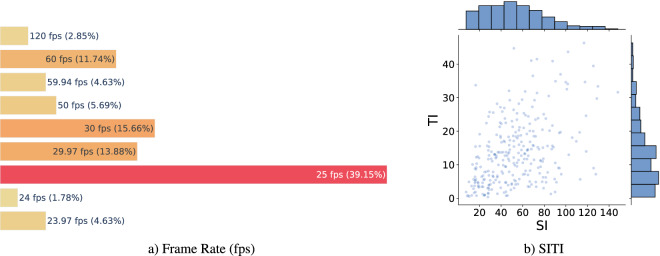
Frame rate and SITI distribution of selected videos.

Figure 2 presents statistics pertaining to the original videos. Notably, the average video size is approximately 8 gigabytes. The smallest video is around 10 megabytes while the largest is approximately 230 gigabytes. Similar variability can be observed in other statistics as well. The average duration of the videos is 29.625 seconds. The shortest video has a duration of only 2.525 seconds, while the longest video spans 888 seconds. On average, the videos consist of 921.69 frames. The frame count ranges from a minimum of 65 frames to a maximum of 21312 frames. The videos possess an average frame rate of 36.262 frames per second (fps). The frame rates in the videos vary from a minimum of 23.97 fps to a maximum of 120 fps. The distribution of frame rates across the videos is depicted in Fig. 3a), indicating that 25 fps is the most frequently utilized frame rate, accounting for 39.15% of the videos. This is followed by 30 fps, utilized in 15.66% of the videos, and 29.97 fps, used in 13.88% of the videos. Finally, the considered videos cover a wide range of the Spatial and Temporal Information (SITI) indexes as shown in Fig. 3b).

From the previous analysis we can see the great variability of the videos databases considered to develop this dataset. This variability is a positive aspect because helps reducing the bias on the type of video.

### Video curation

Due to the extensive number of databases used in this study, we encountered a few instances of repeated videos that had to be discarded. While the occurrences were not significant in number, we thoroughly reviewed all videos to ensure that none were overlooked.

Despite the claims of all the sites regarding the lossless nature of the videos, we performed a validation. This validation process consists on checking the following information:

- YUV video files: Information from database reference webpage.
- Y4M video files: Information from database reference webpage and/or flag “Compression mode” in video metadata is lossless.
- MP4 video files: Videos where quantizer value is 0.
- MOV video files: Information from database reference webpage and/or videos with ProRes RAW codec with “HQ” flag activated.
- MKV video files: Video flag “CodecID” in video metadata is V_UNCOMPRESSED or FFV1 (lossless intra-frame video coding format).

During this phase, we were not able to determine, by using *ffprobe* and *mediainfo* tools or the information provided in their webpage, if the source videos of the Waterloo IVC 4K Video Quality Database^15^ indeed underwent some form of encoding with information loss. Consequently, all the videos from that database were discarded.

Another aspect we had to take into account was the presence and accuracy of metadata. Some videos, due to their container format, lacked metadata, requiring us to gather the following characteristics from their respective databases’ web pages:

- Frame rate: number of frames per second.
- Chroma sampling: it refers to the fact that color information can be subsampled more aggressively than luminance as it does not produce perceptual loss.
- Bit depth: number of bits used to represent luminance and chrominance, usually 8 or 10.

In a few cases, even when metadata was present, it contained conflicting information compared to what was published on the web pages from which we obtained the videos. In such instances, we estimated the correct metadata through trial and error. Table 2 displays a selection of the information found in the video metadata. For example, we discovered that not all videos contained information regarding chroma sampling, and some had incorrect frame rate values. These errors were resolved in the next step by incorporating the appropriate information.

**Table 2:** Information present in video metadata.

File Extension	# videos	Chroma Sampling	# videos	Bit Depth	# videos
**mkv**	96	**4:2:0**	102	**8**	18
**mov**	15	**4:2:2**	16	**10**	82
**mp4**	86	**4:4:4**	1	**Total**	100
**y4m**	35	**Total**	119		
**yuv**	49				
**Total**	281				

After removing all the videos that failed to meet the specified requirements, a total of 281 videos in 4K resolution were ultimately included, as indicated in Table 3.

**Table 3:** Number of videos and 2-second segments per database.

Database	CDVL	AVT	SJTU	XIPH	IVC	MCML	UGC	UVG	TVD	CL	BLEN	Total
**# videos**	7	8	15	25	15	10	88	16	86	9	2	281
**# segments**	879	34	74	119	87	50	837	60	86	1028	811	4065

### Video homogenization

Taking into account the diverse range of containers, chroma sampling, and bit depths utilized (as depicted in Table 2), the next step involved homogenizing these characteristics for all videos included in our collection.

While preserving the original duration and frame rate of each video, we performed encoding using YUV 4:2:0 chroma sampling and 8-bit depth. The encoded videos were saved in the mp4 container format. To ensure lossless encoding, we utilized the *ffmpeg* tool v5.1.2 (http://www.ffmpeg.org/) and employed the H.264 encoder with the Constant Rate Factor control mode set to CRF = 0.

Furthermore, during this stage, we ensured the correction or addition of any incorrect or missing values in the video metadata. Finally, appropriate options were included in each case to rectify the discrepancies.

### Video temporal splitting and spatial downscaling

After the homogenization process, each video was split into 2-second segments. The selection of this segment size is based on a previous study^16^, which indicates that a good choice for persistent connections (very common in current scenarios) is equal to 2 seconds. Table 3 presents the number of segments obtained for each video database considered, resulting in a total of 4065 unique segments. After the splitting phase, each segment was scaled with vertical resolutions of 240, 360, 480, 720, 1080, 1440 and 4K. This gives a total of 28455 segments. All these tasks were performed using the *ffmpeg* tool.

From this point onward, a bifurcation occurred with the goal of creating the datasets presented in this paper. On one hand, the segments were encoded with different CRF values to obtain the following quality metrics: Video Multi-Method Assessment Fusion (VMAF) (https://netflixtechblog.com/toward-a-practical-perceptual-video-quality-metric-653f208b9652), Peak Signal-to-Noise Ratio^17^ (PSNR), and Structural Similarity Index Measure^17,18^ (SSIM). On the other hand, considering that codecs exploit spatial and temporal redundancies, several video features related to spatial information, color information, and temporal information were extracted.

### Quality Datasets

Based on information provided by the authors of the *ffmpeg* tool, who suggest that encoding videos with a CRF value of 17 or 18 provide visually lossless videos that remain nearly identical to the originals, despite not being technically lossless (see https://trac.ffmpeg.org/wiki/Encode/H.264), we decided to initially focus on CRF values between 7 and 51 (considering our extensive use of low resolutions, we decided to lower the minimum CRF parameter to 7).

For an end user, it is virtually impossible to discern any perceptible difference between the same video with two distortions (*V*_1_, *V*_2_) with VMAF scores that are only one unit apart, such as *V*_1_ with a VMAF of 99 and *V*_2_ with a VMAF of 100. Kah *et al*.^19^ have demonstrated that achieving a Just Noticeable Difference (JND) requires a VMAF difference greater than or equal to 2. Hence, we made the decision to exclusively employ CRF values falling within the predefined range that produced VMAF scores equal to or exceeding 1 or below or equal to 99.

The following steps were undertaken to perform all the encodings (see Algorithm 1): for segments with a resolution greather or equal than 1080, we initiated the encodings with CRF=51 (as it is extremely uncommon to obtain a VMAF score less than or equal to 1 with this CRF for these resolutions), and subsequently decreased it by 1 until reaching 7 or achieving a VMAF score greater than or equal to 99. Once either condition was met, we proceeded to the next segment. For other resolutions, encodings began with CRF=7 (as it is highly unusual to obtain a VMAF score greater than or equal to 99 with this CRF for these resolutions), and then increased by 1 until reaching 51 or attaining a VMAF score less than or equal to 1, at which point we moved on to the next segment.

The quality metrics for each encoded segment were calculated using the *ffmpeg*+*libvmaf* v2.3.0 tool. The VMAF calculation for resolutions up to 1080 were computed using the json model vmaf_v0.6.1.json, while the json model vmaf_4k_v0.6.1.json was used for 1440 and 4K resolutions.

### Features Datasets

By utilizing the *ffprobe* tool (http://www.ffmpeg.org/) with the *signalstats* and *entropy* filters, as well as employing the Python *siti* package v1.4.5 (https://github.com/slhck/siti) in accordance with ITU-T Recommendation P.910 (november 2021, https://handle.itu.int/11.1002/1000/14828), we have extracted the collection of features presented in Table 4.

**Table 4:** Set of features obtained with the *ffprobe* tool and Python *siti* package.

**Features per frame obtained with the** ***ffprobe*** **tool**	
Y, U, V	luminance (Y) and chrominance values (U, V)
SAT	saturation value
HUE	value for hue
nEY, nEU, nEV	normalized graylevel entropy in histogram of luminance (Y) and chrominance channels (U, V)
**Features per frame obtained with the Python** ***siti*** **package**	
SI	spatial information
TI	temporal information

Using the Python *numpy*^20^ v1.22.4 and *scipy*^21^ v1.10.0 packages, we calculated the mean, standard deviation, kurtosis, and skewness for each of the mentioned features for every segment. Additionally, we considered video scene detection (with the *scdet* filter of the *ffprobe* tool, thus it has been computed programmatically and it can yield false positives or negatives) and frame rate. In total, 42 features per video segment were computed.

## Data Records

This section provides a detailed description of the dataset that has been made publicly accessible on figshare repository^22^.

### Algorithm 1

Encoding of segments.

We have generated two CSV files per each resolution, one for the quality metrics and the other one for features extracted from the 2-second segments. The naming convention used for each case is *Quality_res*.csv and *Features_res*.csv, respectively, where *res* = {240, 360, 480, 720, 1080, 1440, 4K}.

The quality files consist of the following columns: **file**, which contains the video file names from which the segments were created; **chunk**, an integer value representing the index of each segment within the original video (specifically, the n-th segment corresponds to the time interval [n × 2, (n+1) × 2] seconds); **crf**, an integer value indicating the CRF used during the coding of the segment; **res**, an integer value denoting the resolution of the segment; **vmafmean**, a float value representing the average VMAF across all frames in the segment; **psnrmean**, a float value representing the average PSNR across all frames in the segment; and **ssimmean**, a float value representing the average SSIM across all frames in the segment. Therefore, the quality datasets consist of a total of 7 columns.

The features files also contain three columns with the same purpose as the quality files: **file,** **chunk**, and **res**. This allows for data linkage between the two datasets. Additionally, the features files include the following columns: **sceneChange**, which has a 0 value if no scene change has been detected and 1 otherwise; and **fr**, which provides float values indicating the frame rate of the segment. Finally, as indicated in Table 4, for each feature obtained with the *ffprobe* tool and the Python *siti* package, four columns are provided to display the average, standard deviation, kurtosis, and skewness across all frames in the segment (all float values). The column names consist of the acronym of the feature followed by the strings *mean*, *std*, *kurt*, and *skew* to indicate the respective statistics. For example, if the feature is luminance (abbreviated as Y), the dataset will include four columns named **Ymean,** **Ystd,** **Ykurt**, and **Yskew**. In total, the feature datasets consist of 45 columns.

Considering the number of segments created (refer to Table 3), each of the features files contains exactly 4,065 rows. However, in the case of the quality files, as calculations were not performed for all possible CRF values (refer to the Quality Datasets subsection), the number of rows varies for each file, although all of them have more than 150,000 rows. By combining all the datasets, it is possible to create a single dataset with over 1,000,000 rows and 49 columns.

It is important to note that the tables in the features files may contain NaN (Not a Number) values. The presence of these values does not indicate errors. Skewness and kurtosis are computed by dividing by a power of the standard deviation, therefore if a feature has a zero or nearly zero (10^−15^) standard deviation, it is possible to obtain a NaN value for the skewness or the kurtosis.

Understanding that tables without NaN values are preferred, we have also made available the same datasets without NaN values. In the features files, we simply removed the rows that contain any NaN value. As a consequence, in the quality files, the entries related to the removed rows in the features files (i.e., all the rows of the corresponding quality file that contain rows with the same values in the file and chunk columns) have also been removed. The names of these new datasets are the same as the original datasets, with the addition of the string “_withoutNaN”. For example, you can find datasets named “Features_360_withoutNaN.csv” or “Quality_360_withoutNaN.csv”.

Finally, we provide a CSV file, “datasetVideoSources.csv”, that contains the detailed list of video sequences used in this dataset. It consists in three columns: **name**, a string with the name of the video; **dataset**, a string with the acronym of the database; and **source**, a string with the DOI or URL of the database.

## Technical Validation

If we were to extract the same statistics from the segments as shown in Fig. 2, we would observe the following:

- File Size: It depends on the resolution of the segments. Higher resolution segments generally have larger file sizes due to the increased amount of visual information.
- Duration: The duration of all segments remains the same, which is 2 seconds.
- Frame Count: The frame count of each segment is twice the frame rate. Since the duration is fixed at 2 seconds, segments with higher frame rates will have more frames.
- Frame Rate Distribution: The distribution of frame rates varies significantly. This is because the duration of the original videos plays a role in determining the frame rate distribution of the segments. Longer videos result in multiple segments with the same frame rate as the original video, while shorter videos produce fewer segments.

**Fig. 4:**
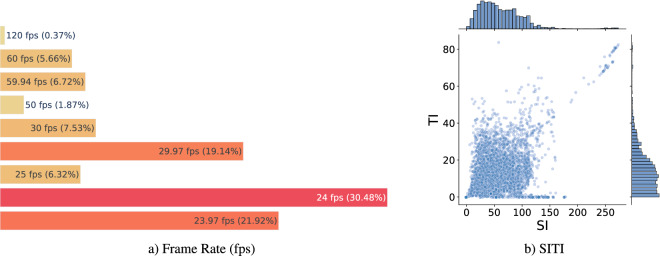
Frame rate and SITI distribution of 2-second video segments.

Figure 4a illustrates the resulting distribution. The minimum and maximum frame rates remain unchanged at 23.97 fps and 120 fps, respectively. However, the frame rate of 24 fps is now the most commonly used, accounting for 30.48% of the segments. It is followed by 23.97 fps, representing 21.92% of the segments, and 29.97 fps, making up 19.14% of the segments. The previously dominant frame rate of 25 fps among the original videos now ranks as the sixth most used frame rate at 6.32%, representing a difference of 32.83 percentage points. These changes occur because the original videos, with varying durations, were transformed into 2-second segments, resulting in a shift in the frame rate distribution. Figure 4b shows the distribution of SITI of all the segments in the dataset, observing a higher variability and a larger range of SITI values.

**Fig. 5:**
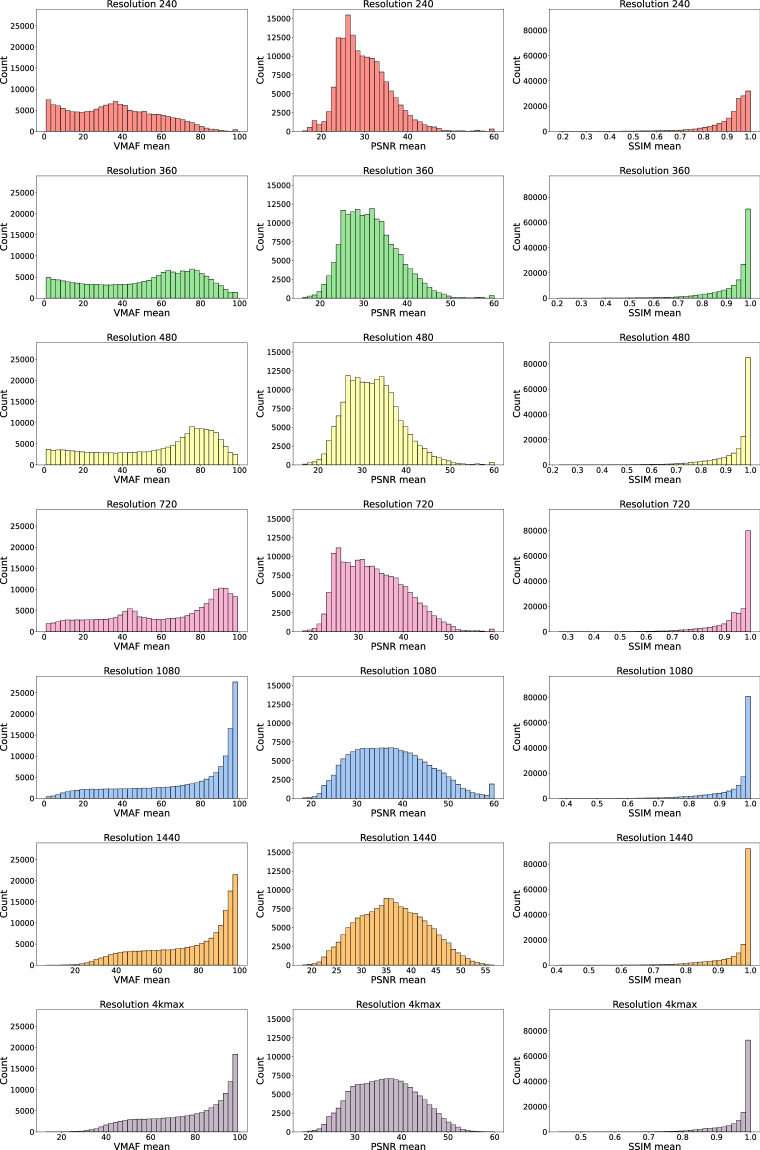
Distribution of VMAF, PSNR and SSIM quality metrics per resolution.

The following analysis pertains to the Quality datasets with all the values. Figure 5 illustrates the distribution of values for each quality metric across different resolutions. The first column represents the distribution of VMAF values, the second column shows the distribution of PSNR values, and the third column displays the distribution of SSIM values.

As the video resolution increases, an upward trend can be observed in VMAF values, with most values below 40 at 240 resolution and most values above 80 at 4K resolution. The figures illustrate the transition in the other resolutions.

In terms of PSNR values, at 240 resolution, the majority of values are concentrated around 27, with a pronounced peak of over 15,000 counts. As the resolution increases, the peak becomes less pronounced, and more values reach approximately the same maximum counts. The concentration of values shifts towards higher PSNR values. For instance, at 360 resolution, the maximum counts remain close to 12,000 around value 29. At 480 resolution, the maximum counts are similar, but set around 32. For 720 resolution, except for a few cases, the maximum counts are below 10,000, around value 32. Finally, at 1080 resolution, the counts stay below 7,000, with many of them around the value of 37. For the 1440 and 4K resolutions, the data continues to accumulate around the value of 37, exceeding 7500 at their respective peaks.

For all resolutions, the SSIM values concentrate towards 1.0, with the concentration becoming more pronounced as the resolution increases. This trend is evident in the averages obtained for each resolution: 0.912 for 240 resolution, 0.932 for 360 resolution, 0.939 for 480 resolution, 0.942 for 720 resolution, 0.953 for 1080 resolution, 0.960 for 1440 resolution, and 0.963 for 4K resolution.

The behaviour of each metric is predictable considering that VMAF is an objective video quality metric based on a machine learning model that simulates human visual perception. PSNR measures the signal-to-noise ratio by comparing the original video to the compressed video and quantifying the amount of added distortion. SSIM is a metric used to measure the structural similarity between two images and is considered a perceptual measure of quality. Among these metrics, VMAF is the most sensitive to changes in video quality and is considered the best predictor of the quality perceived by the viewer^23,24^.

Regarding the features, it is important to note that for the majority of them, the resolution used does not significantly affect the obtained information. This has been demonstrated in the work of Micó-Enguídanos *et al*.^25^ The conclusions reached in that work are as follows:

- For Y, U, V, and SAT, the fidelity of the data remains consistent regardless of the resolution.
- For nEY, nEU, nEV, TI, and HUE, there are minor differences that become more noticeable as the resolution decreases. However, these differences still maintain a clear correspondence between the data obtained at each resolution.
- For SI, the disparities between the data obtained at different resolutions are substantial, resulting in a loss of any linear dependency among them.

**Fig. 6:**
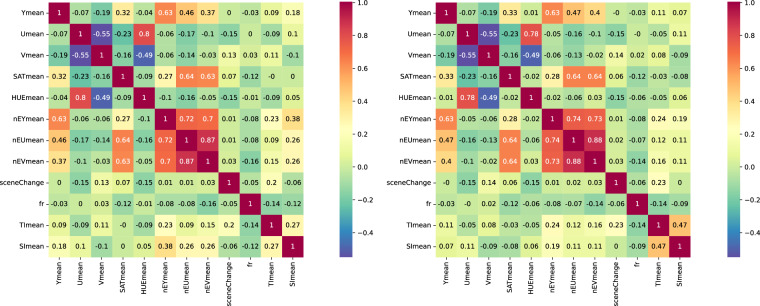
Pairwise Pearson Correlation of Features: 240 resolution (left) and 1080 resolution (right).

As a representative example, Fig. 6 illustrates the Pairwise Pearson Correlation between all features for resolutions 240 and 1080. It can be observed that for any pair of features excluding SI, the obtained correlation values are nearly identical regardless of the resolution. However, with the SI feature, significant variations in the correlation values can be observed.

Moreover, Fig. 6 also demonstrates that the selected features, for the most part, do not exhibit a strong correlation with each other, indicating a lack of linear dependency among them. Only the pair U and HUE, as well as any pair among nEY, nEU, and nEV, display correlation values above 0.7, signifying a strong correlation between them. The majority of other feature pairs have correlation values below 0.3, indicating a practically negligible correlation between them.

**Fig. 7:**
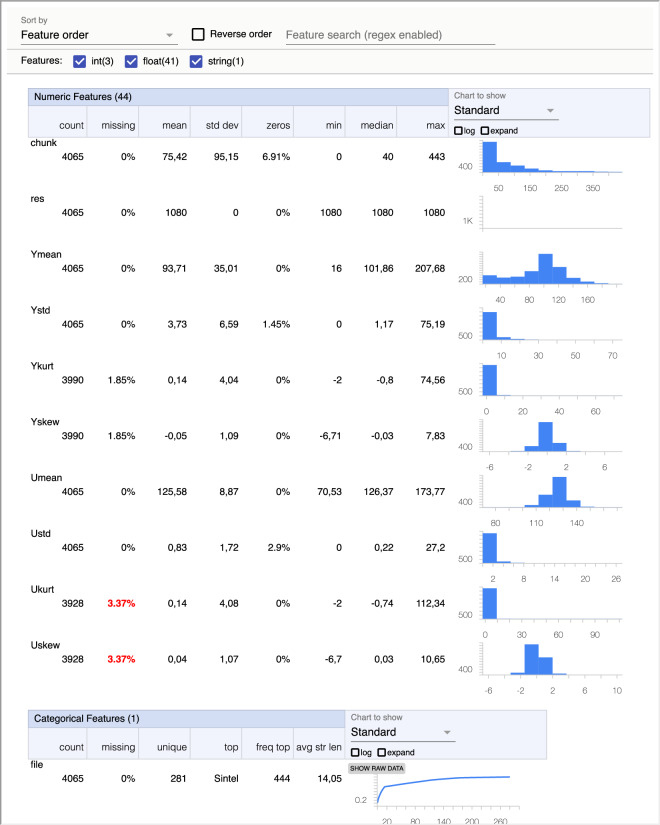
Example of statistics obtained with TFDV for the features without removing NaN values.

The latest analysis conducted on the datasets involved the use of TensorFlow Data Validation (TFDV, https://www.tensorflow.org/), a tool that provides descriptive statistics and detects anomalities in the data. TFDV enabled us to gain insights into the data by presenting important characteristics such as mean, median, standard deviation, and visual representations of value distributions. Figure 7 serves as an example, showcasing the information obtained from the *Features_1080.csv* dataset. It is worth noting the presence of NaN values (as missing values) in the calculations of kurtosis and skewness for the features, as explained in the Data Records section.

## Usage Notes

This dataset offers the possibility to develop new studies that relate video features with quality metrics at several resolutions. As follows, we present some ideas.

This dataset could be used to study the relationships between the features extracted from the video segments with the quality metrics obtained for different CRFs. For example, this dataset could be used to train deep neural networks (DNNs) in order to predict encoding parameters to achieve a target quality. Once the DNN is trained with this dataset, it would be possible to predict the CRF adaptively for each segment of a new video. This idea was successfully applied^25^ using a much smaller dataset with only one resolution.

Furthermore, it allows to compare the quality values obtained in different video segments with similar features (and to find the reason for these differences), or to study to what extent the differences between the segments of the same video affect the calculation of the quality metrics. This could help in the development of new objective quality metrics taking into account segment features.

## Data Availability

Sample Python code, FFmpeg commands, and bash source code used to extract features, compute VQA metrics, handle the datasets, statistical analysis and plots is available at https://github.com/cloudmedialab-uv/VideoCodingFeaturesQualityDataset.
